# Controlling tetramer formation, subunit rotation and DNA ligation during Hin-catalyzed DNA inversion

**DOI:** 10.1093/nar/gkv565

**Published:** 2015-06-08

**Authors:** Yong Chang, Reid C. Johnson

**Affiliations:** 1 Department of Biological Chemistry, David Geffen School of Medicine, University of California, Los Angeles, Los Angeles, CA 90095-1737, USA; 2 Molecular Biology Institute, University of California, Los Angeles, Los Angeles, CA 90095, USA

## Abstract

Two critical steps controlling serine recombinase activity are the remodeling of dimers into the chemically active synaptic tetramer and the regulation of subunit rotation during DNA exchange. We identify a set of hydrophobic residues within the oligomerization helix that controls these steps by the Hin DNA invertase. Phe105 and Met109 insert into hydrophobic pockets within the catalytic domain of the same subunit to stabilize the inactive dimer conformation. These rotate out of the catalytic domain in the dimer and into the subunit rotation interface of the tetramer. About half of residue 105 and 109 substitutions gain the ability to generate stable synaptic tetramers and/or promote DNA chemistry without activation by the Fis/enhancer element. Phe106 replaces Phe105 in the catalytic domain pocket to stabilize the tetramer conformation. Significantly, many of the residue 105 and 109 substitutions support subunit rotation but impair ligation, implying a defect in rotational pausing at the tetrameric conformer poised for ligation. We propose that a ratchet-like surface involving Phe105, Met109 and Leu112 within the rotation interface functions to gate the subunit rotation reaction. Hydrophobic residues are present in analogous positions in other serine recombinases and likely perform similar functions.

## INTRODUCTION

Tight control over any reaction involving severing of DNA strands is of critical importance because of the risk of permanent chromosome damage. This is particularly true of DNA recombination reactions mediated by the serine recombinase family of enzymes (1,2). During the process of DNA exchange, serine recombinases generate double strand cuts at two locations within one or two different DNA molecules, and the cleaved DNA ends are held together by a noncovalent hydrophobic interface between recombinase polypeptides. Moreover, this interface is dynamic, enabling the subunits with their covalently attached DNA ends to rotate about each other to generate the recombinant DNA configuration. Poorly understood mechanisms that are intrinsic to the enzyme must exist to modulate this rotation and thereby allow the recombinases to re-seal the chromosome(s). The present study provides new insights into intrinsic controls over both the formation of the chemically active tetramer and the subunit rotation reaction using the Hin serine recombinase system as the model.

Hin is a member of the DNA invertase subfamily of serine recombinases (3). These enzymes only efficiently catalyze inversion between two specific recombination sites on the same DNA molecule. In the case of Hin, the inversion reaction regulates the expression of alternative flagellin genes in *Salmonella enterica* by switching the orientation of a promoter (4). A related subfamily of serine recombinases is referred to as resolvases because they primarily catalyze deletions (5). Resolvases and invertases share very similar protein structures, although the architectures of the respective synaptic complexes are different (1). Other subfamilies of serine recombinases catalyze other types of DNA rearrangements and have different domain structures. All share a conserved ∼100–130 amino acid residue catalytic domain followed by a long ∼35 amino acid oligomerization helix that functions in synaptic complex assembly and in the chemical and mechanical steps of DNA strand exchange.

**Figure 1.**
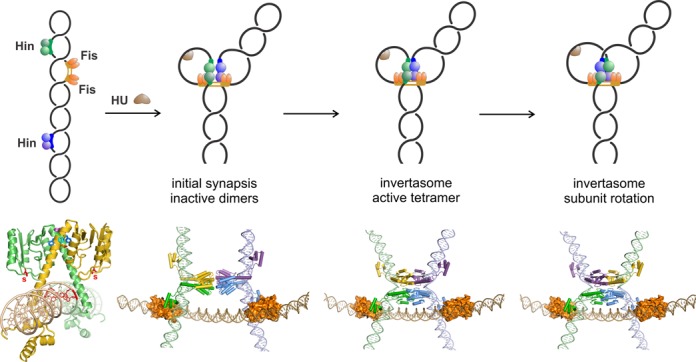
The Hin-catalyzed site-specific DNA inversion reaction (3). Top panels (left to right) schematically depict the reaction pathway. (1) Hin dimers bind to the two *hix* recombination sites and Fis dimers bind to two sites on the recombinational enhancer on a supercoiled DNA molecule. (2) The HU DNA bending protein aids in initial synapsis of the two Hin-bound dimers with the Fis dimers at the enhancer that is located at the base of a plectonemic branch. (3) Formation of an active tetramer that is competent for DNA cleavage and subunit rotation. (4) Supercoiling-directed clockwise rotation of the top pair of Hin subunits; the bottom pair remains associated with the enhancer by Fis and DNA contacts. Bottom panels: structure models of intermediates in the Hin inversion reaction (9,12,34). Left, Hin dimer-*hixL* structure modeled from the γδ resolvase catalytic domain and helix E (PDB: 1GDT) and Hin DNA binding domain (PDB: 1IJW). Red nucleotides mark the core base pairs where DNA cleavage and exchange occurs; S marks the catalytic Ser10 (also in red). Amino acid residues discussed in this paper are colored as in Figure 2. The next three structure models represent the initial synaptic complex of inactive Hin dimers, the active invertasome with a fully assembled tetramer, modeled after γδ resolvase (PDB: 1ZR4), and the Hin tetramer in the invertasome after subunit rotation, respectively. These and other structure images were generated using PyMOL (http://www.pymol.org/).

Serine DNA invertases like Hin are distinguished by the requirement for a remotely positioned recombinational enhancer sequence and its binding partner Fis (3,6–8). During the course of the reaction, Hin dimers bound at the *hix* recombination sites assemble together at the Fis-bound enhancer element (see Figure 1). Synapsis occurs at the base of a plectonemic DNA branch on supercoiled DNA. Within this complex, the inactive Hin dimers become remodeled into a tetramer that is active for DNA cleavage and subunit rotation (9). This Fis/enhancer-dependent remodeling process is arguably the most important regulatory step in the Hin recombination reaction. During the remodeling reaction, dimer contacts that largely involve residues within the oligomerization helix E are replaced by a new set of helix E–helix E contacts between subunits of synapsing dimers. Comparison of X-ray structures of γδ resolvase dimers and tetramers shows that each subunit undergoes a rotation and pivoting about the equivalent of serine 99 in Hin that joins the catalytic domain to the oligomerization helix E (10,11). Newly synapsed subunit pairs (yellow & purple and green & blue subunits in the Figure 1 structure models) are associated through a hydrophobic and ‘flat’ interface that enables rotation of the top pair relative to the bottom pair, which is fixed onto the enhancer through Fis-Hin and Hin-enhancer DNA contacts (9,12). Upon cleavage, the DNA ends are covalently associated with the active site serine via a serine-phosphodiester bond, and translocation of the DNA ends is coupled to rotation of the Hin subunits (13). Most DNA exchange reactions involve only a single 180° rotation, although multiple rotations can occur under certain conditions (14,15). The short DNA length between *hixL* and the enhancer is one mechanism that limits most subunit rotations to a single exchange in the Hin system, but as discussed below, intrinsic features within Hin and other serine recombinases also operate to pause subunit rotation at the correct position for ligation. An intrinsic gating mechanism controlling subunit rotation will prevent drastic losses of DNA supercoils and the formation of complex topologically linked products.

**Figure 2.**
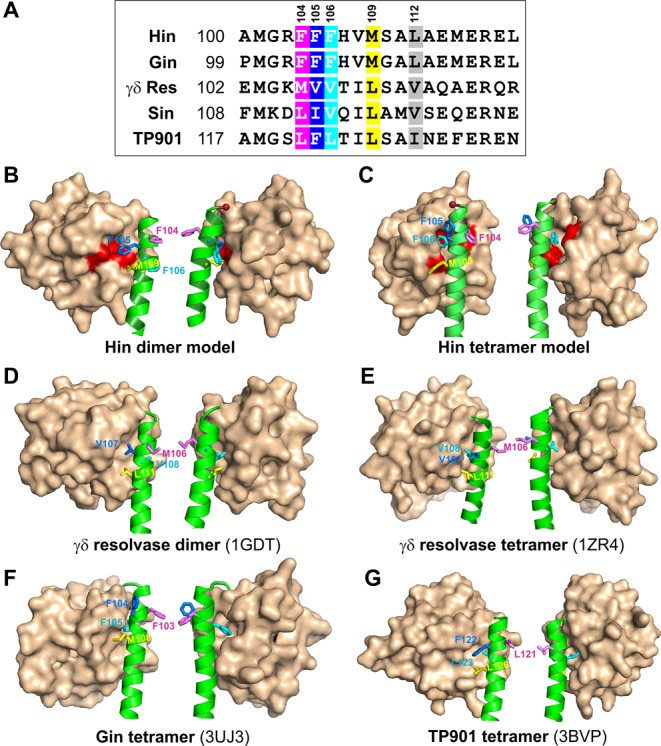
Helix E–catalytic domain interactions. **(A)** Sequence of helix E residues of Hin and other serine recombinases whose structures are available. The Hin phenylalanine triad (residues 104–106), Met109 and Leu112, along with equivalent positioned residues in the other recombinases, are color highlighted. **(B)** Hin dimer model (based on PDB: 1GDT), **(C)** Hin tetramer model (based on PDB: 1ZR4), **(D)** resolvase dimer (PDB: 1GDT), **(E)** γδ resolvase tetramer (PDB: 1ZR4), **(F)** Gin tetramer (PDB: 3UJ3) and **(G)** TP901 integrase tetramer intermediate PDB: 3BVP). In each case the left and right images represent ‘front’ and ‘back’ views of the catalytic domain surface and the N-terminal half of helix E (green). The side chains of Hin residues Phe104, Phe105, Phe106 and Met109 and their equivalents in the other recombinases are color coded as in panel A. The red residues in the Hin catalytic domain mark those where strong hyperactivating mutations have been isolated (Leu64, Leu67, Leu77, Ile78 and Phe88) and the red sphere is hinge residue Ser99 connecting helix E to the catalytic domain (16).

Screens for Hin mutants that could promote inversion without the Fis/enhancer activating system or induce the host SOS DNA damage response identified a localized cluster of residues within the catalytic domain that were remote from the catalytic site (16). These were modeled to form a cleft within the catalytic domain that is referred to as the Phe88 pocket because of the central role of this phenylalanine in organizing the local structure (Figure 2B and C). Substitutions of Phe88 to Leu, Ile or Val and mutations in five additional surrounding residues to smaller hydrophobic amino acids generated hyperactive Hin mutants. The side chain character of these residues is conserved among serine recombinases, and mutations at some of the analogous residues in the Tn3 and Sin resolvases also lead to hyperactivity (17,18). In the Hin dimer structure model, the aromatic side chain of Phe105, which protrudes from helix E of the same subunit, is inserted into the Phe88 pocket (Figure 2B). In the γδ resolvase dimer X-ray structure, the Val107 side chain is oriented into an analogous, albeit somewhat shallower, cleft (Figure 2D) (11). During remodeling to the active synaptic tetramer, Hin Phe105 is switched out of the Phe88 pocket and the Phe106 side chain is rotated into the pocket (Figure 2C), as confirmed by the recent Gin tetramer X-ray structure (Figure 2F) (19). Likewise, γδ resolvase residue Val108 is translocated into its catalytic domain in place of Val107 during the dimer-to-tetramer conversion (Figure 2E) (10). An X-ray structure of a TP901 serine integrase tetramer captured what is believed to be an intermediate in the formation of the active tetramer; in this structure a phenylalanine in the analogous position as Hin Phe105 (Figure 2A) is inserted into a deep pocket in the catalytic domain in a similar manner as proposed for the Hin dimer (Figure 2G) (20).

The phenotypes of the Hin mutations within the Phe88 pocket combined with the dimer and tetramer structures suggest that the exchange of hydrophobic residues during rotation of helix E relative to the catalytic domain mediates a key control step during the inactive dimer to active tetramer conversion (16). However, other than F105V, altered regulatory mutations at helix E residues 105 and 106 were not obtained in the earlier genetic screens with Hin or other DNA invertases (16,21). Mutations leading to hyperactivity at the equivalent of position 105 have also been isolated in the Tn3 and Sin resolvases (17,18). In the present study we have evaluated the *in vivo* and *in vitro* properties of each amino acid residue at positions 105 and 106. In addition we have examined the effects of substitutions at Met109, whose side chain is predicted to insert into a shallower pocket on the surface of the catalytic domain in the Hin dimer (Figure 2A). A leucine in the resolvase dimer at the position analogous to Hin Met109 also inserts into a pocket in its catalytic domain (Figure 2D). We find that Hin residue 109, like 105 and 106, is extremely sensitive to changes. Different substitutions at 105 and 109 exhibit a remarkable variety of phenotypes, including a ligation defect that has not been observed previously by other Hin mutants. The side chains of residues 105 and 109 are repositioned into the rotation interface during formation of the tetramer. We propose that these residues participate in stacking and interdigitating van der Waals interactions between subunits across the rotating interface that function to stall the subunit rotation reaction at the conformer poised for ligation. Thus, they play critical roles in regulating both the formation of the active tetramer and the subunit rotation reaction. It is likely that similarly positioned hydrophobic residues control formation of synaptic tetramers and function to gate subunit rotation during DNA strand exchange in other serine recombinases.

## MATERIALS AND METHODS

### Mutagenesis and *in vivo* assays of Hin activity

Hin mutations were introduced into pMS571 containing *tacP0-hin* (7) for *in vivo* assays and pRJ1518 (pET11a-*hin*) (22) for protein expression and purification using the QuikChange method. *In vivo* assays of Hin activity were performed as described in Heiss *et al*. (16). Briefly, Hin plasmids were transformed into the *fis^+^* inversion tester strain RJ3635 (F′ *proAB^+^ lacI^qs^ lacPL8 Δlac proA::IS10 recF::kan* λ*fla-lac406 off*) or the *fis* mutant inversion tester strain RJ3945 (RJ3635 *fisΔ(2–26)*) and plated onto fresh MacConkey-lactose agar media containing ampicillin. SOS DNA damage response assays used RJ1657 (F′ *proAB^+^ lacI^q^ Z_U118_* NK8027: *Δ(lac-pro) rpsL thi Δ(gal-λG) P_L_-lacZ CI^434^ pRS7*). Colony color was monitored between 22 and 48 h at 37°. These assays rely on low basal expression of Hin; in the case of the inversion reactions, the Lac repressor contains the non-inducible *lacI^s^* mutation D274N. A number of the residue 105 and 109 Hin mutants, even some that are inactive *in vitro*, exhibited growth defects, particularly when plated on MacConkey media (noted in Tables 1 and 2.).

**Table 1.** *In vivo* DNA inversion and SOS DNA damage response for residues 105 and 106 mutants

F105X mutation	Inversion +Fis^a^	Inversion -Fis^b^	SOS^c^
F (WT)	++	±	±
G	-*^d^	-*	ng^e^
A	-*	-*	ng
V	++	+	+*
L	+++	++	++
I	+++	++	+*
M	+++	++	+*
W	++*	+*	±
Y	+++	±	+
P	-	-	-
S	-	±	-*
T	++	±	+
C	++	±	+
N	+++	+	++
Q	±*	±*	+*
H	-	-	++
D	-	-	-
E	-	-	-
K	-*	-*	-
R	-*	-*	-
**F106 mutations**^f^	-	-	-

^a^Hin-catalyzed DNA inversion assayed using a chromosomal *lacZ* reporter in a *fis^+^* host: (+++) indicates fully red colonies (promoter inversion) on lactose MacConkey media developed within 24 h, (++) red colonies between 24 and 28 h (Hin-wt ∼26 h), (+) red colonies between 28 and 36 h, (±) some red color (trace of inversion) after 36–40 h and (-) colonies remained white after 40 h. The no Hin vector control was white after 45 h.

^b^Hin-catalyzed DNA inversion in a *fis* mutant host: (++) indicates red colonies on lactose MacConkey media developed within 30 h, (+) red colonies between 30 and 36 h, (±) some red color developed after 36–40 h and (-) colonies remained white up to 48 h like the vector control.

^c^Hin-activated host SOS response assayed using a chromosomal λ*P_L_-lacZ* reporter: (++) indicates red colonies developed on lactose MacConkey media between 26 and 30 h, (+) red colonies between 30 and 36 h, (±) red colonies after 40 h as observed for Hin-wt and (-) remained white at 48 h as observed for the vector control.

^d^Asterisk indicates score is tentative due to slow growth, often with heterogeneous colony sizes.

^e^ng indicates extremely poor or no growth on the MacConkey media.

^f^F106 mutations include all amino acid residues other than phenylalanine (wt). All mutants exhibited no activity except F106Y, which gave a trace of +Fis inversion (light red colonies after 38–40 h).

**Table 2.** *In vivo* DNA inversion and SOS DNA damage response for residue 109 mutants

M109X mutation	Inversion +Fis^a^	Inversion -Fis^b^	SOS^c^
M (WT)	++	±	±
G	-	-	±
A	±*^d^	±*	ng^e^
V	±*	±*	ng
L	±*	±*	ng
I	±*	±*	ng
F	+++	+	-
W	+++	+	±
Y	+	-	++
P	-	-	-
S	-	-	-
T	-	-	-
C	±*	±*	ng
N	-	-	-
Q	+*	+*	++
H	±	-	++
D	-	-	-
E	-	-	-
K	-	-	++
R	-	-	++

^a^Hin-catalyzed DNA inversion assayed using a chromosomal *lacZ* reporter in a *fis^+^* host: (+++) indicates fully red colonies (promoter inversion) on lactose MacConkey media developed within 24 h, (++) red colonies between 24 and 28 h (Hin-wt ∼26 h), (+) red colonies between 28 and 36 h, (±) some red color (trace of inversion) after 36–40 h and (-) colonies remained white after 40 h. The no Hin vector control was white at 45 h.

^b^Hin-catalyzed DNA inversion in a *fis* mutant host: (++) indicates red colonies on lactose MacConkey media developed within 30 h, (+) red colonies between 30 and 36 h, (±) some red color developed after 36–40 h and (-) colonies remained white up to 48 h like the vector control.

^c^Hin-activated host SOS response assayed using a chromosomal λ*P_L_-lacZ* reporter: (++) indicates red colonies developed on lactose MacConkey media between 26–30 h, (+) red colonies between 30 and 36 h, (±) red colonies after 40 h as observed for Hin-wt and (-) remained white at 48 h as observed for the vector control.

^d^Asterisk indicates score is tentative due to slow growth, often with heterogeneous colony sizes.

^e^ng indicates extremely poor or no growth on the MacConkey media.

### Hin purification and *in vitro* assays

Hin mutants were expressed and partially purified as described in Heiss *et al*. (16) except that typically only 100 ml of culture were employed and lysates were generated using a Bioruptor (Diagenode). A representative panel of Phe106 mutant preparations displayed on a sodium dodecyl sulphate-polyacrylamide gel electrophoresis (SDS-PAGE) gel is shown in Supplementary Figure S1A. *In vitro* inversion assays were performed using pMS551 ((7), Supplementary Figure S2) as described (16,23); reactions in the absence of Fis included 15% ethylene glycol, which we have noted previously with other hyperactive Hin mutants generally result in activities that more closely match their *in vivo* phenotypes (16). Inversion rates accounted for the back reaction (24); for slow reactions, rates were calculated over 1–10 min time frames. Initial cleavage rates were typically calculated from 1 min reactions after restriction digestion, but for very active mutants, rates were calculated from 30 s reactions and extrapolated to 1 min, even though most products formed by 30 s. Therefore, the per minute cleavage rates given for some of the highly active mutants are underestimates. Electrophoretic mobility shift assays (EMSA) were performed as described (16,25); for synaptic complexes, gels contained 10% glycerol. Reactions in the experiments shown in Figures 4 and 5 contained 25% ethylene glycol, but inclusion of ethylene glycol was not necessary for synaptic complex formation by these mutants.

**Figure 3.**
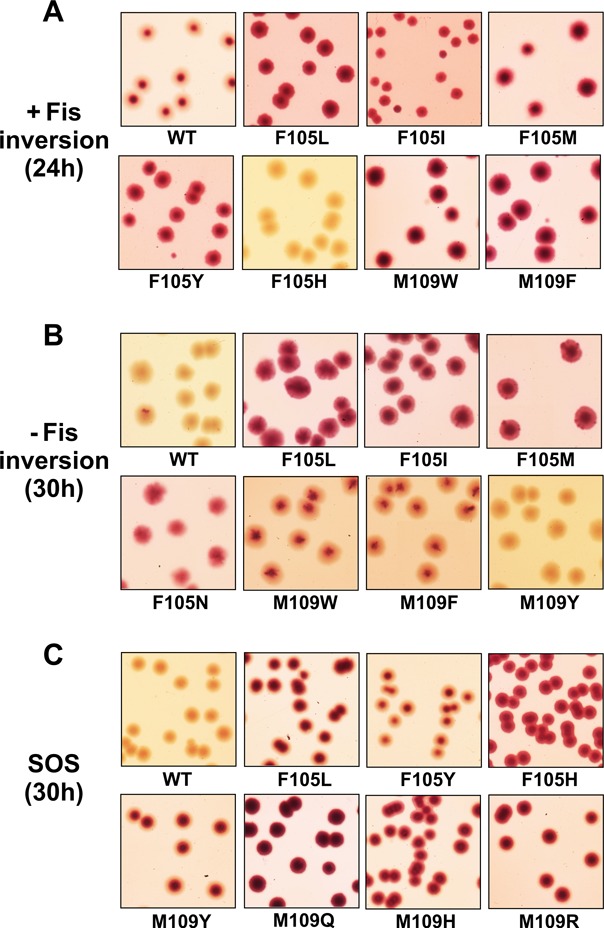
*In vivo* Hin activity assays. **(A)** Hin-catalyzed DNA inversion assays in *fis^+^ Escherichia coli* cells containing the H inversion region controlling *lacZ* transcription. Transformations with Hin mutant plasmids were plated on MacConkey-lactose media with antibiotic. Representative images were obtained after 24 h incubation. **(B)** Inversion assays in *fisΔ(2–26) E. coli* cells. Images were obtained after 30 h incubation. **(C)** Hin induction of the SOS DNA damage response by *E. coli* cells containing λ*CI^434^ P_L_-lac*. Images were obtained after 30 h incubation on MacConkey-lactose media with antibiotic after Hin plasmid transformation. All images were adjusted equally for brightness and contrast and enlarged ∼50%.

**Figure 4.**
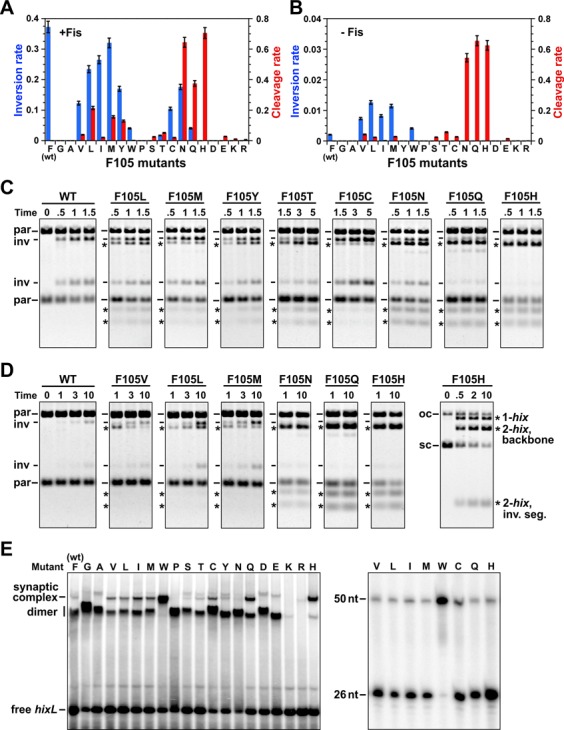
*In vitro* activities by Hin-Phe105 mutants. **(A** and **B)**. Rates of DNA inversion (blue bars, inversions/molecule/min) and *hix* cleavage (red bars, cleavages/molecule/min), with standard deviations given, in the presence (A) and absence (B) of Fis. **(C)** Representative *in vitro* DNA inversion assays on supercoiled pMS551 after digestion with Pst I and Hind III performed in the presence of Fis. Parental (par) and inversion (inv) product bands are denoted along with the products of Hin cleavage at *hixL1* and *hixL2* (asterisks). See Supplementary Figure S2 for illustrations of the substrate plasmid and reaction products. Hin incubation time is in minutes. **(D)** Representative *in vitro* DNA inversion assays performed without Fis. The panel on the right is without digestion with restriction enzymes; only Hin-generated DNA cleavages, which excise the invertible segment from the vector backbone or linearize the plasmid (cutting at only one *hix* site), are revealed. **(E)** Representative EMSA showing Phe105 mutant complexes on a 50 bp *hixL* fragment (left panel). The synaptic complex band was extracted in the presence of SDS, digested with proteinase K and subjected to denaturing PAGE (right panel). The 26 nt band is the product of Hin cleavage.

**Figure 5.**
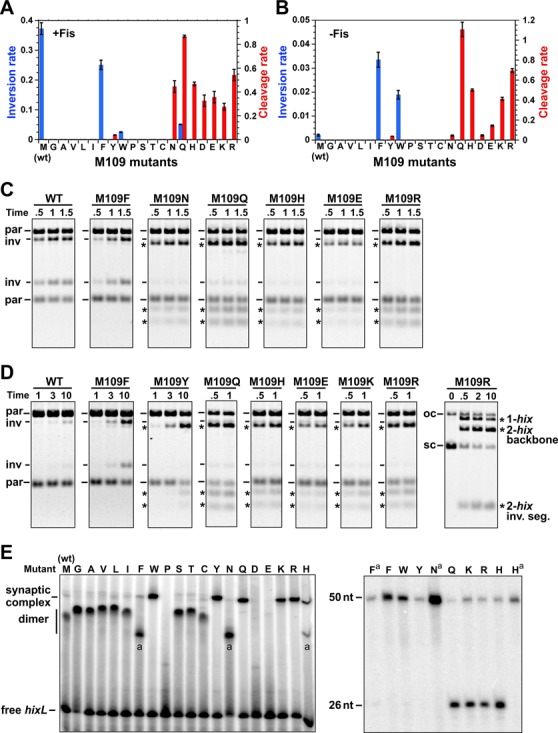
*In vitro* activities by Hin-Met109 mutants. **(A** and **B)** Rates of DNA inversion (blue bars, inversions/molecule/min) and *hix* cleavage (red bars, cleavages/molecule/min), with standard deviations given, in the presence (A) and absence (B) of Fis. **(C** and **D)** Representative *in vitro* DNA inversion assays in the presence and absence of Fis, respectively. See Figure 4 legend for details. **(E)** Representative EMSA showing Met109 mutant complexes on a 50 bp *hixL* fragment (left panel). Bands labeled (a) are believed to represent Hin dimer complexes in an altered conformation. Right panel is a denaturing gel evaluating Hin-catalyzed DNA cleavage (26 nt band) within the native gel complexes. F^a^, N^a^ and H^a^ are from the complexes labeled (a) in the native gel.

**Figure 6.**
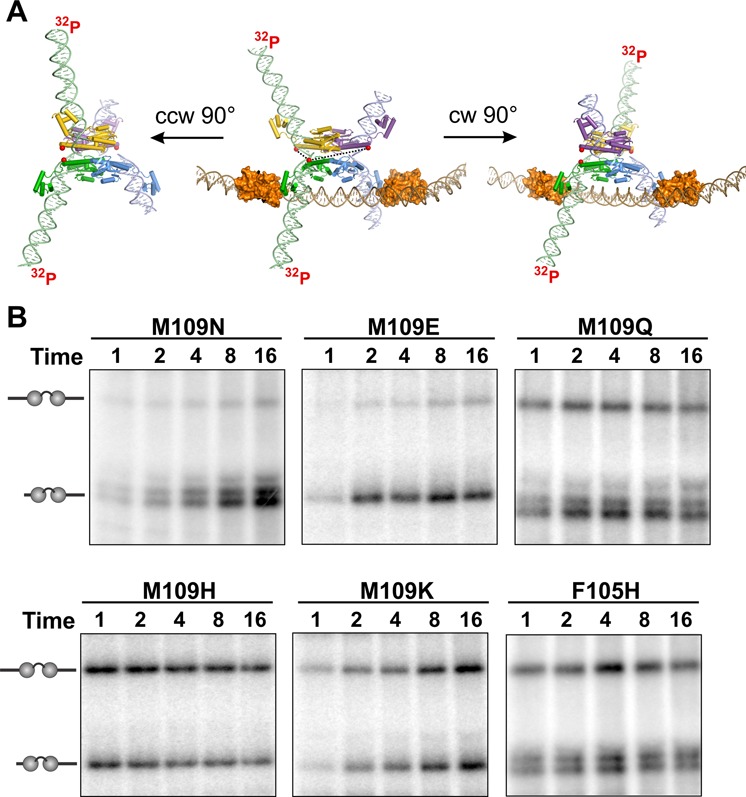
Subunit rotation and tetramer stability evaluated by site-directed crosslinking. **(A)** Crosslinking assay. Hin mutants coupled with Q134C were incubated with pRJ2330 under standard inversion conditions and subjected to a 20 s crosslinking reaction with BMOE at various times. After quenching the Hin and crosslinking reactions, the products were digested with EcoR1, which cleaves 56 bp from the center on each side of *hixL-1* and immediately adjacent to each side of *hixL-2*. The central panel shows the modeled structure of the Hin-cleaved invertasome after EcoR1 digestion and end-filling with ^32^P-dATP before subunit rotation. Cys134 SH (red spheres) between subunits are ≥50 Å apart. A ∼90° clockwise subunit rotation (right panel) positions Cys134 from the purple Hin subunit that is covalently linked to the short purple DNA for crosslinking to the green subunit that is covalently linked to the long green labeled DNA. A single rotation will be favored in the Fis/enhancer-associated invertasome complex in part because the DNA segment between *hixL-1* and the enhancer is only 155 bp. A ∼90° counter clockwise subunit rotation (left panel) positions Cys134 from the yellow Hin subunit that is covalently linked to the long labeled green DNA appropriately for crosslinking to the green subunit covalently linked to the long green labeled DNA. Counter clockwise rotation can occur without an associated enhancer (12). The long–long DNA crosslinking product could also be generated from multiple clockwise rotations without the enhancer. **(B)** Phosphorimager of crosslinking time courses. At the designated Hin reaction times (min) the different mutants were subjected to 20 s crosslinking reactions. These reactions were performed in the presence of Fis. M109N/Q134C and M109E/Q134C DNA cleavage reactions are strongly stimulated by Fis whereas M109Q/Q134C, M109H/Q134C, M109K/Q134C and F105H/Q134C reactions exhibit moderate to little stimulation by Fis. See Supplementary Figure S3 for comparison of crosslinking profiles from reactions performed with and without Fis. The Hin crosslinked products are denoted with the schematic long–long and long–short DNA segments. Overrepresentation of long–short products (M109N and M109E) is indicative of a single clockwise 90° rotation. Double bands are due to incomplete denaturation of Hin because of the brief heat treatment used to minimize hydrolysis of the phosphoserine bond (12). Note that under the DNA inversion reaction conditions used in these experiments, no wild-type Hin (containing Q134C) crosslinking products would be obtained because no DNA cleavage products accumulate. Crosslinking efficiencies (percent of total Hin-DNA cleavage complexes that were crosslinked) at the 16 min time point are as follows: M109N 78.5%, M109E 73.5%, M109Q 61%, M109H 72%, M109K 83.5% and F105H 66%. Hin-Q134C (wt) gave 65% crosslinked products under EDTA+ethylene glycol reaction conditions (see also (12)).

Crosslinking reactions measuring subunit rotation and tetramer stability were performed using pRJ2330 as described ((12); see also Figure 6 legend), except that standard inversion buffer conditions (20 mM Hepes, pH 7.5, 100 mM NaCl, 10 mM MgCl_2_) were used during the Hin incubation and crosslinking steps. BMOE (bis-maleimidoethane, 8 Å spacer, Pierce-Thermo Scientific) was used as the crosslinker, and the Hin mutants containing Q134C were pre-reduced by overnight incubation at −20° with 12 mM Tris(2-carboxyethyl)phosphine. Crosslinking reactions (20 s) were quenched with 40 mM DTT and 0.04% diethylpyrocarbonate and then digested with EcoR1 and labeled at the 3′ ends with α-^32^P-dATP using klenow.

## RESULTS

### Properties of amino acid substitutions at Phe105 and Phe106

#### *In vivo* activities

To probe the regulatory importance of the phenylalanine switch mechanism whereby insertion of Phe105 into the catalytic domain Phe88 pocket is replaced by insertion of Phe106, we generated all amino acid substitutions at these two positions. The Hin mutants were first evaluated for their ability to catalyze inversion *in vivo* with and without Fis. For these assays we used the λ*fla406* reporter, which contains the *Salmonella enterica* Typhimurium H inversion region with an inactivated *hin* gene controlling transcription of *lacZ* (24). The starting prophage contains the invertible segment in the off orientation, and introduction of *hin-wt* on a plasmid produces red colonies on MaConkey-lactose media within 26 h as a result of promoter inversion (see Figure 3A). Almost half of the position 105 mutants exhibited little-no detectable inversion in *fis^+^* cells (Table 1). These include charged and some of the polar residues. On the other hand, most Phe105 mutants containing hydrophobic residues had significant activity with leucine, isoleucine, methionine, tyrosine, as well as asparagine substitutions exhibiting apparent inversion rates that were faster than wild type (Figure 3A). Whereas Hin-wt is essentially dependent upon Fis for inversion *in vivo*, a number of the Phe105 mutants with smaller or more flexible hydrophobic amino acid substitutions exhibited substantial inversion in *fis* mutant cells (Figure 3B, Table 1). Leucine, isoleucine, methionine substitutions were particularly strong, but valine, tryptophan and asparagine also promoted Fis-independent inversion. F105V was previously identified as a mild hyperactive mutant in Gin and Hin (16,21).

We also evaluated whether the Phe105 mutants induced the SOS DNA damage response when introduced into a host containing *lacZ* under the control of the λP_L_ promoter (Figure 3C and Table 1). Half of the position 105 substitutions induced SOS. These include each of the hydrophobic amino acid residue substitutions that generated Fis-independent inversion plus tyrosine, threonine, cysteine, asparagine, glutamine and histidine. F105L, N and H are particularly strong, and notably, F105H exhibits no inversion even though it generates a robust SOS response.

All substitutions at Phe106 inactivated Hin for DNA inversion, except for tyrosine, which exhibited very weak *in vivo* activity. Partially purified preparations of each of the Phe106 mutants were competent for dimer binding to the *hixL* recombination site (Supplementary Figure S1B), but none formed tetramers in our synaptic complex assay (see below; data not shown), and with the exception of tyrosine, none promoted detectable inversion. Fis-activated *in vitro* reactions with Hin-F106Y produced inversions at 10% the rate of wild type, which is somewhat higher than expected from the weak *in vivo* activity. The results with residue 106 suggest that no amino acid residue other than phenylalanine and tyrosine (weakly) is capable of forming active synaptic complexes.

#### *In vitro* activities of Phe105 mutants

Each of the Phe105 substitution mutants was partially purified and assayed for DNA inversion in the presence and absence of Fis. Fis/enhancer-activated *in vitro* inversion rates generally followed their *in vivo* activities (Figure 4A and C) with the hydrophobic residues typically supporting moderate to high inversion rates, albeit none of the mutants exceeded the wild type. Leucine and methionine exhibited the highest Fis-independent inversion rates *in vitro*, but the rates of these along with the other mutants that displayed clear Fis-independent inversion *in vivo* were less than expected from their *in vivo* activities (Figure 4B and D). We also observed that *in vitro* inversion rates by hyperactive mutants containing changes within the Phe88 pocket were less than expected from their *in vivo* activities (16).

The most striking *in vitro* property of many of the Phe105 mutants was the accumulation of DNA molecules cleaved at their *hix* sites that occurred under both +Fis and -Fis conditions (Figure 4A–D). These are illustrated in the examples in Figure 4C and D where the Hin reactions were subsequently subjected to restriction digestion to reveal both the orientation of the invertible segment and double strand cleavages at the *hix* sites (asterisks). *Hix* cleavage products had previously only been detected under EDTA-ethylene glycol reaction conditions, not under standard magnesium-containing inversion conditions. F105H generated exclusively unligated DNA products at rates, with or without Fis, that were greater than the Fis-activated wild-type inversion rate; Figure 4D (right hand panel) shows a reaction with F105H without subsequent restriction digestion, highlighting the Hin-induced cleavages at the *hix* sites. F105Q behaves similarly to F105H except that it generates very small amounts of inversion. Time course reactions illustrate that some of the mutants slowly converted *hix*-cleaved molecules to ligated inversion products. Examples include +Fis reactions with F105L, M and N in Figure 4C and -Fis reactions with F105V, L and M in Figure 4D. In the case of F105N and Q, formation of ligated inversion products only occurred in the presence of Fis. The slow rate or absence of detectable ligation by the Phe105 mutations *in vitro* fits with their *in vivo* SOS induction phenotypes.

Hin-wt forms synaptic tetramers extremely inefficiently in the absence of the Fis/enhancer system (25). The phenylalanine switch mechanism predicts that amino acid residues at position 105 whose side chains are less tightly associated within the Phe88 pocket may exhibit enhanced formation of tetramers. Each of the Phe105 mutants was tested for its ability to form synaptic tetramers in the absence of Fis that were stable during gel electrophoresis. The mutant proteins were incubated with ^32^P-labeled 50 bp *hixL* fragments in the presence of high ethylene glycol and EDTA, conditions that have been shown to promote synaptic tetramers by other Hin hyperactive mutants (16,25), and subjected to native PAGE. As shown in Figure 4E, residue 105 mutants with tryptophan, histidine and glutamine substitutions formed high levels of synaptic tetramers, and those with valine, leucine, isoleucine, methionine and cysteine exhibited weaker tetramer formation. The *hix* sites in all the synaptic complexes except F105W were mostly cleaved upon extraction from the gel in the presence of SDS and thus in a catalytically active conformation (Figure 4E, right panel). F105K and R did not form detectable DNA complexes by the gel mobility shift assay even though they catalyzed a very low cleavage reaction in solution. In summary, eight of the position 105 substitutions showed enhanced tetramer formation by the EMSA assay.

### Properties of amino acid substitutions at Met109

#### *In vivo* activities

The side chain of Met109, which is located one helical turn below Phe105 on helix E, is also predicted to associate with the catalytic domain of the same subunit of the dimer and be repositioned into the rotating interface of the tetramer (Figure 2B and C). Therefore, we asked whether substitutions at residue 109 would exhibit phenotypes similar to those observed for residue 105.

*In vivo* inversion assays showed that substitutions at residue 109 were less tolerated than observed for residue 105 (Table 2). Only phenylalanine or tryptophan substitutions displayed efficient inversion; these appeared to promote inversion faster than wild type in the presence of Fis and were also active in the absence of Fis (Figure 3A and B). Tyrosine and glutamine were the only other substitutions that gave appreciable inversion *in vivo*. Five of the position 109 mutants generated a strong SOS DNA damage response. These include M109H, K and R, which exhibited little to no detectable inversion. Interestingly, M109Y generated a robust SOS response, but M109F and M109W did not significantly induce SOS.

#### *In vitro* activities of Met109 mutants

M109F catalyzed efficient *in vitro* inversion with Fis, but M109W was less active than expected from the *in vivo* assays (Figure 5A and C). Both catalyzed significant Fis-independent inversion over Hin-wt (Figure 5B and D). As observed for Phe105 mutants, many of Met109 mutants accumulated primarily or exclusively *hix*-cleaved products under standard *in vitro* inversion conditions with or without Fis (Figure 5A–D). These include those with charged amino acid residues and large polar residues (histidine, asparagine, tyrosine and glutamine). With the exception of a small amount of ligated inversion products for M109Q in the presence of Fis, none of these appeared to support any ligation. Remarkably, whereas M109F generated almost entirely ligated inversion products, M109Y generated exclusively DNA cleaved products (e.g. Figure 5D). Taken together, half of the substitutions at residue 109 promoted Fis-independent catalytic activity with most of these leading to exclusively cleaved DNA molecules.

All residue 109 mutants except substitutions by proline, aspartic acid and glutamic acid supported normal DNA binding to *hix* as evaluated by EMSA assays (Figure 5E). Even though M109D and E did not form DNA complexes stable to gel electrophoresis, they were able to cleave *hix* under inversion reaction conditions, as noted above. M109W, Y, Q, K and R generated exclusively synaptic tetramers under all protein concentrations tested. It is possible that some of these mutant proteins may be tetrameric in solution, although experiments to test this have not been conclusive. The DNA within gel-isolated synaptic complexes formed by M109Q, H, K and R are cleaved at the centers of the *hix* sites, but those formed by M109W and Y are not cleaved (Figure 5E, right panel). In summary, six of the Met109 mutants generate synaptic tetramers with DNA fragments in the absence of Fis. We conclude that Met109 is an important residue controlling the dimer–tetramer conversion in a manner related to Phe105.

M109N, most of M109F, and variable amounts of M109H complexes migrated aberrantly fast and contained uncleaved DNAs (Figure 5E). We believe these to be Hin dimer complexes as judged from the similarity of their migrations to those of Hin dimer complexes formed on DNA fragments in which one half of the *hix* site is replaced with random sequence DNA (data not shown). The altered migrations presumably reflect differences in the conformation of the Hin dimer–DNA complexes. Each of these mutants exhibits hyperactivity *in vitro* as measured by Fis-independent inversion or cleavage, but M109N was not active *in vivo*.

### Subunit rotation by the ligation-defective mutants

Time-resolved site-directed crosslinking experiments showed that the ligation-defective mutants are competent for subunit rotation and remain as stable tetrameric complexes after DNA cleavage. These reactions were performed by first incorporating the Q134C mutation into a subset of Phe105 and Met109 mutants. Crosslinking at Cys134 by BMOE (8 Å spacer) requires either a ∼90° clockwise rotation, which will nearly exclusively occur under Fis/enhancer-activated conditions, or either a clockwise or counterclockwise rotation, which can occur under Fis/enhancer-independent conditions (Figure 6A) (12). A dissociated tetramer will not support crosslinking. Hin was added to supercoiled pRJ2330, which contains one *hix* site flanked by EcoR1 sites 56 bp from the Hin cleavage sites and the other *hix* site flanked by EcoR1 sites 13 bp from the Hin cleavage sites. pRJ2330 contains only 155 bp between *hixL1* and the enhancer so subunit rotation in Fis/enhancer-activated reactions is typically limited to a single clockwise exchange (12,14,15). After incubation with Hin for 1–16 min, BMOE was added, and the crosslinking and Hin reactions were quenched after 20 s. The products were digested with EcoR1 and subjected to end fill-in reactions with ^32^P-labeled dNTPs whereby only the 56 bp covalently linked DNAs will label with DNA polymerase. Single clockwise rotations are indicated by crosslinked Hin synaptic dimers containing a covalently linked labeled 56mer and an unlabeled 13mer after SDS-PAGE. Single counterclockwise rotations are indicated by crosslinked Hin synaptic dimers containing two labeled 56 mers (these products could also be formed by even numbers of multiple clockwise exchanges). The crosslinking experiments in Figure 6 were performed under standard inversion conditions containing Mg^2+^. Previous crosslinking experiments that followed subunit rotation have been performed under reaction conditions employing EDTA and 30% ethylene glycol, which were required to prevent re-ligation of the *hix* sites and thus trap the Hin-(^32^P)DNA covalent linkage.

Figure 6B shows crosslinking results for three different classes of ligation-defective mutants. M109N/Q134C and M109E/Q134C promote largely Fis-dependent *hix* cleavages. These proteins efficiently generated crosslinked products indicative of primarily single ∼90° clockwise rotations in the presence of Fis (Figure 6B), and as expected, generated very low amounts of crosslinked products without Fis (Supplementary Figure S3 and data not shown). M109Q/Q134C and M109Y/Q134C catalyze DNA cleavage independently of Fis, although are still further activated by Fis. In Fis-activated reactions, products indicative of single clockwise rotations predominated for these mutants (Figure 6B and Supplementary Figure S3). The lower amount of Fis-independent reaction promoted by M109Y/Q134C generated equal amounts of 56–56 nt and 56–13 nt products, consistent with both counterclockwise and clockwise rotations (Supplementary Figure S3). Likewise, M109H/Q134C, M109K/Q134C and F105H/Q134C, which exhibit nearly equal rates of cleavage with or without Fis, give nearly unbiased crosslinking profiles. In all cases, crosslinking efficiencies are robust, typically 60–80% of the Hin-DNA cleavage complexes at the 16 min time point (Figure 6 legend), which provides strong evidence that the tetrameric synaptic complexes are maintained during the course of the experiment. Moreover, mechanisms invoking exchange of synaptic dimers between different complexes would be incompatible with the Fis-activated reactions that generate primarily crosslinked products reflecting single clockwise rotations (e.g. M109N/Q134C and M109E/Q134C).

## DISCUSSION

We initially set out to test the phenylalanine switch mechanism controlling Hin activity whereby the aromatic side chain of Phe105 on helix E rotates out of a pocket on the surface of its own catalytic domain and is replaced by the Phe106 side chain during the dimer–tetramer remodeling step. Insertion of the Phe105 ring into the Phe88 pocket of the catalytic domain is proposed to play a prominent role in stabilizing the inactive dimer conformation (Figure 2B), and likewise, insertion of the Phe106 ring into the Phe88 pocket is proposed to be required for formation of the active tetramer (Figure 2C). We also tested whether Met109 on helix E performs a similar regulatory role as Phe105. As elaborated below, comprehensive analyses of amino acid substitutions at these three positions are consistent with their proposed roles. Other serine recombinases where structural information is available contain similar structural motifs, but with different identities of the key hydrophobic residues (Figure 2). It is therefore likely that the intrasubunit connections identified here generally control formation of chemically active synaptic tetramers by this class of recombinases.

Unexpectedly, a large number of Hin residue 105 and 109 mutants accumulated unligated DNA products during standard inversion reaction conditions, a phenotype only previously observed when EDTA and high concentrations of ethylene glycol are present. Because these residues are relocated onto the surface of the rotating interface in the active tetramer, this phenotype led us to examine the features of the interface that may be responsible for pausing rotation at the appropriate conformer to enable ligation. Control of subunit rotation and the role of Phe105 and Met109, together with Leu112, in facilitating the ligation step is also discussed below. Our findings with Hin point to an intrinsic gating mechanism for regulating the subunit rotation reaction that is likely to be shared by other serine recombinases.

Hin proteins containing over 10 different amino acid residues at position 105 exhibit gain-of-function phenotypes as recognized by their ability to catalyze Fis-independent inversion and/or *hix* cleavage or SOS induction. Eight of these exhibit enhanced formation of synaptic tetramers in the absence of Fis as compared with Hin-wt containing phenylalanine. The smaller aliphatic residues leucine, methionine, isoleucine and valine, which may associate more dynamically with the Phe88 pocket, result in Fis-independent inversion. As shown most clearly from *in vitro* reactions without Fis, most of these mutants initially accumulate cleaved DNA that slowly ligates into inverted products (Figure 4D). The large polar residues histidine, glutamine and asparagine result in robust Fis-independent cleavage activity. Hin-F105H and Q are nearly completely defective in ligation, whereas F105N generates a small amount of ligated recombinant products when Fis is present. Tryptophan, which might be predicted to be too large to comfortably insert into the Phe88 pocket, results in formation of exclusively synaptic tetramers. Even F105Y exhibits a mild hyperactive phenotype *in vivo*, although it remained Fis-dependent. These phenotypes are consistent with the native Phe105–Phe88 pocket interaction performing a prominent role in stabilizing the inactive dimer conformation.

The Met109 side chain is predicted to insert into a shallow pocket of its catalytic domain in the inactive dimer (Figure 2B). Ten of the residue 109 substitution mutants exhibit gain-of-function properties *in vivo* and/or *in vitro*, with six displaying enhanced formation of synaptic tetramers without Fis. Only the Hin mutants M109F, M109W, M109Y (*in vivo*) and M109Q (weak) are able to catalyze complete recombination. Large polar and charged residues typically result in robust Fis-independent DNA cleavage without ligation. Conversely, position 109 residues with small side chains result in catalytically inactive proteins. We conclude that the intrasubunit connection by Met109 with the catalytic domain also plays an important regulatory role in maintaining the inactive dimer state.

All substitutions at residue 106 were catalytically inactive, with the exception of tyrosine that displayed weak inversion activity. This is not unexpected because insertion of Phe106 into the Phe88 pocket is predicted to be critical for stabilizing the active tetramer. We attempted to isolate second site suppressors of several 106 mutants that would support inversion and obtained H107Y coupled with F106L. H107Y is our strongest hyperactivating mutation and has been isolated multiple times both alone and as suppressors of other helix E mutations (16,23,25). We believe that the tyrosine substitution at position 107 stabilizes the tetrameric conformation through interactions of the tyrosine rings between synapsed subunit pairs (16); stabilization of the Sin tetramer by a mutation at the analogous residue has also been proposed based on its crystal structure (26). The suppression by H107Y is thus consistent with a defect in tetramer formation by F106L. The activity of F106L/H107Y also implies that at least a leucine substitution at residue 106 is not causing a major disruptive conformational change in the protein, even though it blocks Hin activity subsequent to DNA binding when present by itself.

### Gating of subunit rotation and the DNA ligation reaction

A hallmark of most of the chemically active residue 105 and 109 mutants is the accumulation of unligated products. The mutant tetramers must be altered in such a way as to allow DNA cleavage but not ligation. The efficient intersubunit crosslinking (Figure 6 and Supplementary Figure S3) demonstrates that the tetramer structures are intact and fully competent for subunit rotation. Given that the mutant residues are not proximal to the active site but rather buried within the rotation interface, an attractive hypothesis is that normal pausing of subunit rotation at the conformer poised for ligation is disturbed. A defect in gating of the subunit rotation reaction would thereby lead to inefficient or even complete failure of the ligation step.

We have previously shown that Hin normally undergoes a single 180° subunit exchange reaction to generate unknotted recombinant (inverted) products upon ligation (12,14,15). If ligation cannot occur because of a mutation with the core residues that prevents base pairing, a second 180° rotation follows, and most of the time ligation then occurs to restore the parental primary DNA sequence. The short (100 bp) DNA segment between the enhancer and *hixL* functions to inhibit processive rotations because of the torsional strain of winding of DNA during subunit rotation. Nevertheless, even with large DNA segments between the *hix* sites and enhancer, the overwhelming majority of reactions (>90–95%) ligate after a single subunit exchange (14,15). In addition, recent single-DNA molecule experiments show that the Fis-independent Hin mutant H107Y relaxes braided DNAs in discrete steps whereby rotation events are temporally separated by seconds to minutes (B. Xiao, R. C. Johnson and J. F. Marko, unpublished data). These observations imply a gating mechanism intrinsic to the Hin tetramer structure that functions to pause subunit rotation at 180° intervals.

**Figure 7.**
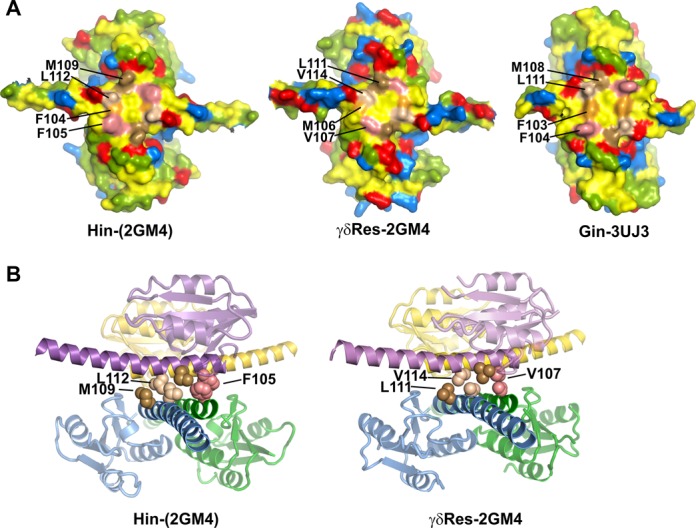
The subunit rotation interface. **(A)** Surface view facing the rotation interfaces of one rotating dimer of the respective tetramers. Only the catalytic domains and a portion of the E helices extending laterally are shown. In general, hydrophobic residues are yellow, polar residues are green, basic residues are blue and acidic residues are red. Specific residues within the rotating interfaces are differentially colored and labeled. The numbering of γδ resolvase residues is shifted by +2 and Gin residues by −1 relative to Hin. The γδ resolvase image is from PDB: 2GM4, Gin is from PDB: 3UJ3 and Hin is from a model based on 2GM4. The rotating surface of γδ resolvase structure PDB: 1ZR4 and the Hin model based on that crystal structure are nearly identical to the respective images shown here. **(B)** View looking into the rotating interface (E helices) of the Hin-2GM4 model and γδ resolvase 2GM4. Side chains of residues that interdigitate or stack on each other as discussed in the text are rendered as van der Waals spheres. The catalytic domains are slightly transparent. The subunit pairs within the Gin tetramer X-ray structure have undergone partial rotation (26°) and therefore do not exhibit these interactions (not shown) (19).

Inspection of the rotational interfaces of tetrameric γδ resolvase X-ray structures and the Hin tetramer models that were based on resolvase reveals that the surfaces are not completely flat (Figure 7A). Rather, several large hydrophobic residues generate protrusions that either interdigitate with each other across the interface or directly stack on each other generating a large common van der Waals surface area. The most prominent of these interfacial residues for Hin are Phe105, Met109 and Leu112; the corresponding resolvase residues are Val107, Leu111 and Val114, respectively. The side chains of Hin residues Met109, Leu112 and Phe105 interdigitate and the aromatic rings of Phe105 stack on each other (Figure 7B). These prominently contribute to a peak of van der Waals surface area overlap at the ‘DNA-aligned’ conformer that is oriented appropriately for ligation (see Supplementary Figure S7 in reference (12)). We propose that these residues form a ratchet to pause subunit rotation in the conformation to initiate ligation. Different amino acid residues substituted at positions 105 and 109 would likely disrupt the ratchet and thereby compromise ligation.

A ratchet mechanism can explain an unresolved concern of the subunit rotation reaction by serine recombinases: why does the protein swivel not create topological problems, including massive chromosome relaxation, knotting, and catenation, and a high incidence of chromosome breaks? Where evaluated, all members of the small serine recombinase family, including the γδ/Tn*3* and Sin resolvases and Hin and Gin invertases, primarily catalyze single-rotation DNA exchanges (14,15,27–30), and all contain hydrophobic residues on the surface of the rotation interface at the equivalent position of Hin Phe105, Met109 and Leu112 (Figures 2 and 7). Topological analyses with serine integrases have given mixed results: studies on ϕC31 (31), Bxb1 (31,32) and A118 (S. Mandali and R. C. Johnson, unpublished data) imply primarily single-rotation DNA exchange reactions, but another study on Bxb1 reports essentially ungated subunit rotations (33). Sequence alignments of the helix E regions of some of the more distantly related serine integrases are ambiguous, but most show hydrophobic residues at the three analogous positions.

A set of charge interactions between acidic residues Asp94/Asp95 and basic residues Arg121/125 were suggested to stabilize the DNA-aligned conformer of γδ resolvase (10). One set of analogous residues, Asp93 and Arg123, is present in Hin and other serine DNA invertases. Both of these residues seem to be playing important roles early in the Hin reaction because non-conservative substitutions strongly inhibit catalytic activity, and in the case of R123C, block synaptic complex formation (34,35) (Y. Chang and R. C. Johnson, data not shown). Hin-D93N promotes weak DNA inversion without an apparent ligation defect (Y. Chang and R. C. Johnson, data not shown). Although we cannot rule out a role of these residues in controlling subunit rotation in Hin, no evidence currently exists to support such a function.

### Hin Phe105 and Met109 mutants as targeted DNA cleaving enzymes for genetic engineering

Finally, we note that the Phe105 and Met109 mutants that robustly cleave the 26 bp *hix* sites have properties reminiscent of restriction enzymes, and, as such, could be exploited as a tool for genetic engineering. These are distinguished from restriction enzymes in that the catalytic mechanism is through nucleophilic attack by an activated serine that results in covalent joining to the protein, as opposed to a hydrolysis reaction. Nevertheless, they efficiently generate double strand breaks at long DNA targets whose sequence recognition is well defined (36,37).

## SUPPLEMENTARY DATA

Supplementary Data are available at NAR Online.

SUPPLEMENTARY DATA
