# Supplementary material for: Controlling tetramer formation, subunit rotation and DNA ligation during Hin-catalyzed DNA inversion
Source: Nucleic Acids Res. 2015 Jun 8;43(13):6459–72. doi: 10.1093/nar/gkv565 (PMC4513852; doi:10.1093/nar/gkv565)
Supplement: SUPPLEMENTARY DATA [file supp_43_13_6459__index.html]

Controlling tetramer formation, subunit rotation and DNA ligation during Hin-catalyzed DNA inversion — Controlling tetramer formation, subunit rotation and DNA ligation during Hin-catalyzed DNA inversion — SUPPLEMENTARY DATA 

# Controlling tetramer formation, subunit rotation and DNA ligation during Hin-catalyzed DNA inversion

## SUPPLEMENTARY DATA

- SUPPLEMENTARY DATA
