# Supplementary material for: Controlling tetramer formation, subunit rotation and DNA ligation during Hin-catalyzed DNA inversion
Source: Nucleic Acids Res. 2015 Jun 8;43(13):6459–72. doi: 10.1093/nar/gkv565 (PMC4513852; doi:10.1093/nar/gkv565)
Supplement: SUPPLEMENTARY DATA [file supp_gkv565_nar-01248-m-2015-File010.pdf]

Supplementary Data

Controlling tetramer formation, subunit rotation, and DNA ligation during Hin-catalyzed DNA inversion

Y. Chang and R. C. Johnson

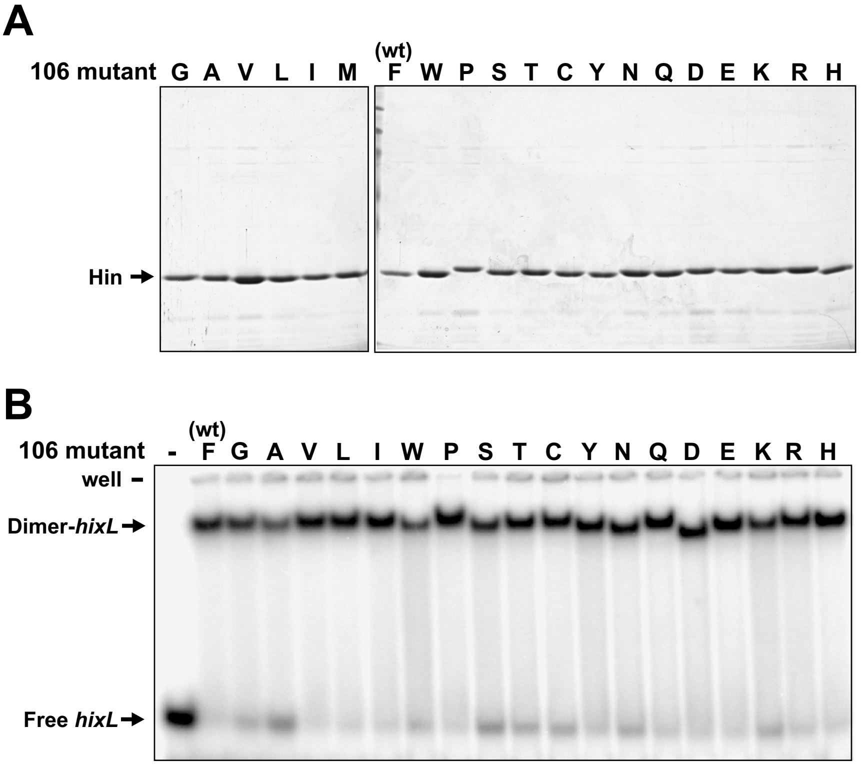

Figure S1. Phe106 mutant protein preparations. A. SDS-PAGE of the set of mutant proteins stained with coomassie blue. B. Representative EMSA using 20 ng (23 nM) Hin protein and a ^{32}P-labeled 50 bp *hixL* fragment. All Phe106 mutant protein preparations efficiently bind *hix* at about 20 nM; recombination assays were performed using 10 to 100 nM Hin with only F106Y exhibiting any activity.

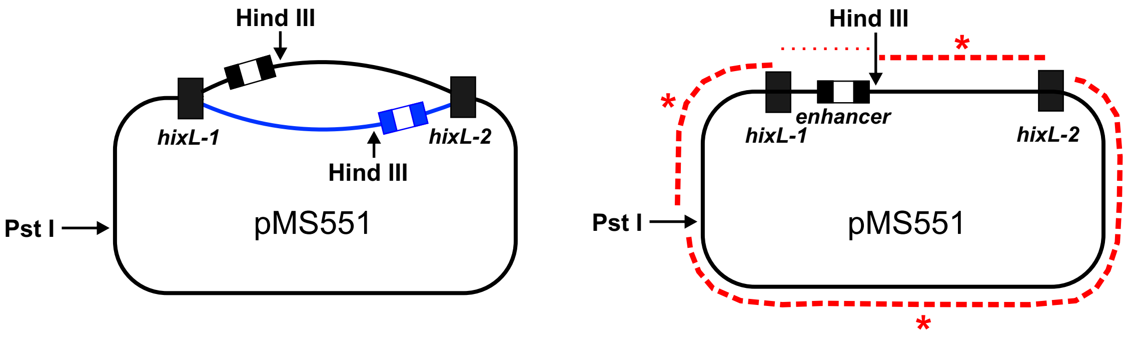

Figure S2. pMS551 substrate used for the in vitro recombination assays (Figure 4 and 5). Left panel depicts the change in the position of the Hind III site relative to the Pst I site after inversion (blue). Right panel depicts DNA fragments (red dashed lines demarked with asterisks in Figures 4 and 5) generated upon Hin cleavage at *hixL-1* and *hixL-2* plus restriction digestion by Pst I + Hind III. The small fragment between *hixL-1* and Hind III (thin red dashed line) is not visible in the agarose gels.

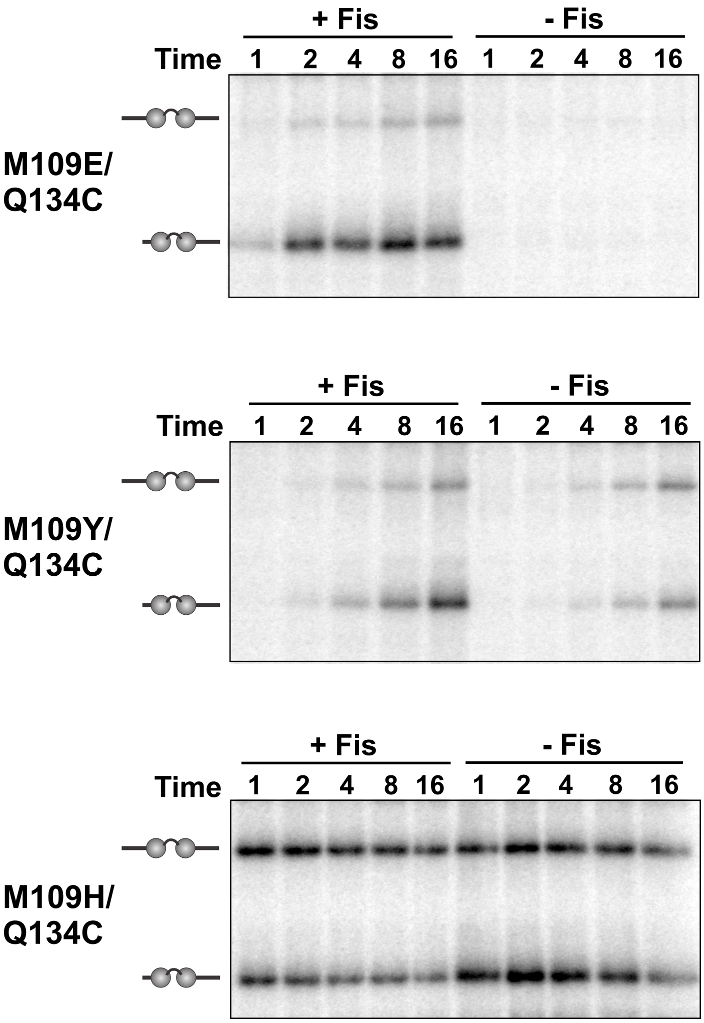

Figure S3. BMOE crosslinking comparing Fis-activated vs. Fis-independent reactions. Reactions were performed as in Figure 6 except that both +Fis and -Fis panels are shown. Hin-M109E/Q134C is strongly stimulated by Fis, Hin-M109Y/Q134C is moderately stimulated by Fis, and Hin-M109H/Q134C is essentially Fis-independent.
